# Inspiring our future psychiatrists: a quality improvement project to optimise the medical student experience in community CAMHS settings

**DOI:** 10.1192/bjo.2021.472

**Published:** 2021-06-18

**Authors:** Brindha Anandakumar, Lois Nunn, Fanchea Daly, Ashleigh Dale

**Affiliations:** South London and Maudsley NHS Foundation Trust

## Abstract

**Aims:**

To improve the structure, quality and experience of medical student placements in Community Child and Adolescent Mental Health Services (CAMHS). To increase the likelihood of pursuing a career in Psychiatry or CAMHS by 50% over their 3 week placement.

**Background:**

There is evidence in the literature of the widely variable medical student experiences when it comes to Psychiatry placements. Medical students from Kings’ College London (KCL) have a 3 week placement in Lambeth Community CAMHS services. Despite this being a good opportunity for learning and development, the feedback from students reports that they often feel lost and were unable to fulfil the potential of the placement. The main challenges reported were identifying beneficial educational experiences and gaining clinically relevant exposure. This exposure includes getting involved beyond observation and following a patient longitudinally. These challenges will likely have a knock-on effect on their attitude towards Psychiatry and overall enjoyment of CAMHS placements when there is already a struggle to recruit trainee Psychiatrists.

**Method:**

A structured and immersive educational placement was designed through consultation with previous students, the multidisciplinary team and the university program directors. This included:

- A new induction
- Having a role in initial assessments of young people
- Formalised medical and psychology teaching
- Communication sessions
- Case discussions in a ‘grand round’ format providing opportunity for end of placement assessment

Feedback was gathered using the Qualtrics analytical software, which was easily accessible through student's mobile devices.

Pre placement questionnaires were used to assess the student's initial level of knowledge, expectations from the placement and motivation or interest in a career in CAMHS. Post placement questionnaires were used to assess any change in the above baseline scores. Brief, online feedback was collected after every clinical activity and was used to assess the interest and utility of each attended session during the placement. The questionnaire feedback was analysed and using these data, adjustments were made to improve the program for the next students in a “Plan-Do Study-Act” quality improvement methodology format. We analysed whether improving placement experience and learning affected students’ interest in careers in Psychiatry.

**Result:**

The Quality Improvement Project is currently on-going and results are pending. So far, there is an improvement in student attendance and engagement following the introduction of induction, structure and active involvement in case management.

**Conclusion:**

The COVID-19 pandemic has resulted in community services having significantly reduced face to face contact, therefore our proposed changes for future cycles include various virtual elements. Ensuring medical students have access to online platforms such as Microsoft teams is vital in ensuring an effective medical student placement can be established Although the change to more remote working has been challenging , it is vital that medical students gain appropriate clinical experience during their Psychiatry placement to support further developments in Psychiatric recruitment.

